# Safety of esthetic procedures in rheumatic patients: single-center survey of patients

**DOI:** 10.1007/s00296-023-05481-5

**Published:** 2023-10-17

**Authors:** Anna Felis-Giemza, Agata Matusiewicz, Anna Wajda, Marzena Olesińska

**Affiliations:** 1 Biologic Therapy Center, National Institute of Geriatrics, Rheumatology and Rehabilitation, Warsaw, Poland; 2 Department of Connective Tissue Diseases, National Institute of Geriatrics, Rheumatology and Rehabilitation, Spartańska 1, 02-637 Warsaw, Poland; 3 Department of Molecular Biology, National Institute of Geriatrics, Rheumatology and Rehabilitation, Warsaw, Poland

**Keywords:** Connective tissue diseases, Esthetics, Tattooing, Cosmetic techniques

## Abstract

The popularity of esthetic medicine is growing every year, also among patients with autoimmune inflammatory rheumatic diseases (AIRD). The objective of this study was to evaluate the safety of esthetic medicine (AM) procedures in patients with AIRD. A semi-structured, anonymous questionnaire regarding rheumatic and concomitant diseases and AM procedures was distributed among adult patients hospitalized in the rheumatology department or attending outpatient clinic in the National Institute of Geriatrics, Rheumatology, and Rehabilitation, Warsaw. The main outcome was the occurrence of an adverse event. A number of 512 patients took part in the survey and 15 were excluded (AM procedure preceded the diagnosis of AIRD). The study group consisted of 497 patients, of whom 47 had undergone AM procedures. The procedures performed included: tattooing (22 patients), piercing (16 patients), hyaluronic acid (7 patients), botulinum toxin (5 patients) injections, laser procedures (6 patients), plastic surgery (4 patients), mesotherapy (3 patients) and others. The vast majority of patients had these performed during remission or low disease activity. 70.2% of patients received treatment with disease-modifying antirheumatic drugs (DMARDs) during the AM procedure, with TNF-alfa inhibitors being the most common (63.6%). Adverse events occurred in 15% of patients. All were mild and transient site reactions. Most patients would like to repeat the AM procedure in the future. The use of esthetic medicine procedures in patients with AIRD, including those treated with biologic DMARDs, was associated with a risk of mild site reactions. Most of the patients expressed satisfaction with the results of the AM procedure.

**Supplementary Information:**

The online version contains supplementary material available at 10.1007/s00296-023-05481-5.

## Introduction

The development of new treatment paradigms for rheumatic diseases in recent years resulted in improvements in patients’ quality of life. Rheumatologists are increasingly dealing with questions about the possibility of using esthetic medicine (AM), plastic surgery, or tattooing. Both men and women go for, more or less, invasive methods to alter their appearance, resulting in the growing popularity of esthetic medicine every year. Unfortunately, these interventions can cause adverse events, even in the hands of experienced professionals. Appropriate selection of patients for the procedure and considering their medical history are extremely important. Some data indicate that the risk of adverse events increases in patients with inflammatory conditions of the skin (psoriasis, rosacea, and skin infections), active connective tissue diseases with skin involvement, or immunosuppressive treatment [1], but data on the safety of patients with autoimmune inflammatory rheumatic diseases (AIRD) are limited and contradictory.

The purpose of this study was to gather information on the performance of esthetic medicine, plastic surgery, and tattooing procedures in patients with AIRD and to assess the incidence of complications following these procedures.

## Materials and methods

This unvalidated survey was drafted by two clinician rheumatologists and revised by a third clinician rheumatologist who decided in any disagreement. The study received ethical approval from the National Institute of Geriatrics, Rheumatology, and Rehabilitation Ethics Committee (KBT-5/10/2022, 08.11.2022). All study procedures were conducted in accordance with the Declaration of Helsinki.

A semi-structured, anonymous questionnaire consisted of 19 closed and open questions regarding demographics, rheumatic and concomitant diseases, and AM procedure (Appendix 1). The main outcome was the occurrence of an adverse event.

We distributed paper booklets among adult patients hospitalized in the Department of Connective Tissue Diseases or attending outpatient clinic—Biological Therapy Center in the National Institute of Geriatrics, Rheumatology, and Rehabilitation, Warsaw, between April 2018 and December 2019. Participation in the survey was voluntary, followed by written consent. Patients completed it personally during the visit. Handwritten answers were collected and entered into database by the authors between April and August 2020.

The inclusion criterium was a diagnosis of AIRD and the exclusion criterium was a history of AM procedure before the diagnosis of AIRD.

GraphPad Prism 8.4.1 has been used to conduct statistical analysis. Numerical data distribution was analyzed using the Shapiro–Wilk test. The Mann–Whitney test, Kruskal–Wallis test, or one-way ANOVA were used for inter-group comparisons. The frequency of categorical data was analyzed using Fisher’s exact test. Statistical significance was set at *p* < 0.05.

## Results

A number of 512 patients took part in the survey and 15 were excluded (AM procedure preceded the diagnosis of AIRD). The study group consisted of 497 patients, of whom 47 had undergone AM procedures (group 2). In group 2, women (*n* = 39; 83%) outnumbered men (*n* = 8; 17%) and the average age of the patient was 36.8 ± 9 years (min. 20, max. 56 years). Among the 450 patients who had never had AM procedures (group 1), there were 254 women (56%) and 196 men (44%) and patients were older (the average age was 48 ± 14 years, min. 18, max. 82 years).

Demographic data and the distribution of diagnoses are shown in Table 1. There were no statistically significant differences regarding place of residence and education between the groups.

**Table 1:** Patients’ characteristics with autoimmune inflammatory rheumatic diseases (AIRD) within study group

	Group 1—patients with AIRD without AM procedure history (*n* = 450)	Group 2—patients with AIRD with a history of AM procedure (*n* = 47)
Education		
Elementary	22 (5%)	2 (4%)
Secondary	211 (47%)	15 (32%)
Higher	216 (48%)	30 (64%)
Answer denial	1	0
Place of residence		
Village	99 (22%)	10 (21.3%)
City < 100,000 inhabitants	121 (27%)	10 (21.3%)
City > 100,000 inhabitants	229 (51%)	27 (57.4%)
AIRD diagnosis^a^		
Axial spondyloarhtritis	154 (34%)	18 (38.5%)
Juvenile idiopathic arthritis	21 (5%)	7 (15%)
Psoriasis arthritis	65 (14%)	3 (6.5%)
Rheumatoid arthritis	197 (44%)	17 (36%)
Systemic lupus erythematosus	2 (0.5%)	1 (2%)
Systemic sclerosis	2 (0.4%)	0
Mixed connective tissue disease	2 (0.4%)	1 (2%)
Still’s disease	2 (0.4%)	0
Sjögren’s syndrome	3 (0.6%)	0
Granulomatosis with polyangiitis	1 (0.2%)	0
Dermatomyositis	2 (0.4%)	0
Idiopathic retroperitoneal fibrosis	1 (0.4%)	0
Concomitant autoimmune diseases^a^		
Hashimoto’s disease	25 (6%)	5 (11%)
Graves’ disease	4 (1%)	0 (0%)
Psoriasis	40 (9%)	4 (9%)
Crohn’s disease	4(1%)	0 (0%)
Ulcerative colitis	8 (2%)	0 (0%)
Vitiligo	5 (1%)	0 (0%)
Diabetes mellitus, type 1	10 (2%)	0 (0%)
Others	1 (0.2%)	0 (0%)

*AIRD* autoimmune inflammatory rheumatic diseases, *AM* esthetic medicine

^a^Patients with more than one diagnosis were present

AM procedures and treatment reported within group 2 are shown in Tables 2 and 3. Only 4 patients had their disease-modifying antirheumatic drug (DMARD) discontinued temporarily due to a planned AM procedure.

**Table 2:** Number of patients with autoimmune inflammatory rheumatic diseases, who underwent esthetic procedures

Tattoo	*n* = 22; 46.8%
Piercing	*n* = 16; 34%
Hyaluronic acid injection	*n* = 7; 14.9%
Botulinum toxin injection	*n* = 5; 10.6%
Laser procedures	*n* = 6; 12.8%
Plastic surgery	*n* = 4, 8.5%
Breast implants	*n* = 2; 4.3%
Perineoplasty	*n* = 1; 2.1%
Facelift	*n* = 1; 2.1%
Mesotherapy	*n* = 3; 6.4%
Others (peptide cosmeceuticals, fat lipolysis)	*n* = 3; 6.4%

**Table 3:** Treatment at the time of the esthetic procedure reported by patients with autoimmune inflammatory rheumatic diseases

TNFi	*n* = 21; 45%
Combination of TNFi and cDMARD	*n* = 3; 6%
Other bDMARD	*n* = 2; 4%
cDMARD	*n* = 3; 6%
Corticosteroid	*n* = 4; 9%
None	*n* = 14; 30%

*bDMARD* biologic disease-modifying antirheumatic drug, *cDMARD* classic disease-modifying antirheumatic drug, *TNFi* tumour necrosis alfa inhibitor

A number of 21 subjects (44.7%) consulted a physician before the procedure, including 14 patients consulting a rheumatologist. Recommendations against the AM procedure were reported by 4 patients (9%) The risk associated with AM procedure was discussed with 27 patients (63%).

Since disease-specific activity scores at the time of the AM procedure were not available, patients were asked to rate disease activity subjectively on a five-point scale. The largest group of subjects underwent AM procedure while remission or mild disease activity (*n* = 39; 66%). Moderate activity was indicated by 12 patients (25.5%) and high activity by 4 patients (8.5%).

Side effects after a procedure affected 7 patients (15%) and included swelling, pain, redness, and bruising. All events were mild and transient and dissipated within 2 weeks; only in 3 cases they required medical consultation.

In group 2, most patients (57.4%, *n* = 27) would like to repeat the AM procedure in the future. In comparison, in group 1, only 37 patients (8%) expressed a desire to undergo such a procedure.

## Discussion

There is a paucity of literature on the safety of AM procedures in patients with rheumatic conditions. However, several aspects concerning this group of patients should be considered.

### Wound healing and blood coagulability

According to the Kluger guidelines (2018), rheumatic diseases such as systemic lupus erythematosus or scleroderma are not a contraindication for tattooing. However, the procedure should not be performed when a disease is active/unstable or in an acute stage of flaring, because skin healing may be delayed, resulting in poor esthetic effect [2]. Chin et al. in their case series reported two of the main complications that can occur in patients with severe connective tissue diseases: delayed donor site wound healing, and delayed hematoma formation [3].

Alternatively, some authors suggested that dermal fillers are not contraindicated in patients in whom wound healing is normal, even though they may have an underlying systemic disease [4, 5]. In addition, Lemperle et al. have not found immunosuppression to delay wound healing, because fibroblasts need an approximately tenfold higher concentration of immunosuppressive drugs to be affected than do immunocytes [4].

### Infections

Immunosuppressive treatment may involve a higher risk of complications such as increased fatigue/diminished stamina during the tattoo session, and increased risk of local or systemic infection [2].

There have been few studies evaluating perioperative clinical features and complications after elective orthopedic surgery in these patients, including the risk of surgical site infection. These studies demonstrated a significant association between early infectious complications and impaired wound healing following orthopedic surgery and treatment with TNF-alfa inhibitors in patients with rheumatoid arthritis [6–8]. It is known that patients with AIRD have been shown to be at higher risk for the development of surgical complications. The highest risk is in those with multiple organ dysfunction or using long-term steroids and immunosuppressive drugs [9]. In plastic surgery, the recommended time of biologic therapy cessation varies between 3 and 5 half-lives of the drug in the patients of low risk, but recent orthopedic recommendations allow to plan surgery after the end of the dose interval (1 to 5 half-lives, respectively) [8, 10].

### Site reactions

Patients with a history of AIRD or receiving immunosuppression should avoid fillers containing copolymers of hydroxy-ethyl-methacrylate (HEMA) and ethyl-methacrylate (EMA), which are non-biodegradable and may cause severe granulomatous reactions [11]. It seems that the most appropriate dermal fillers for such patients are quickly biodegradable or re-absorbable agents [12]. The safety of fillers not approved by medicine agencies has been undermined [12]. A recent study by Koren et al. points to the high level of safety of esthetic treatment with non-permanent fillers in patients with inflammatory rheumatic diseases [13]. Furthermore, two reviews state that patients with autoimmune conditions may be safely treated with a variety of fillers [14, 15]. Cutaneous complications after tattooing occurred in 8.7% patients with psoriasis, with Koebner sign being the most common. No adverse events were observed in patients receiving biologic therapy [16].

### New autoimmune entity development

While some studies have indicated a potential link between esthetic procedures and autoimmune diseases involving the injection of fillers, breast implants, or tattoo ink [4, 5, 17–23], others showed that there was no evidence of an association between AM procedures and AIRD [24]. Controlled epidemiological studies have not shown an actual link between silicone and systemic autoimmune diseases [25–28].

Hence, some authors permit the use of fillers in patients with systemic lupus erythematosus, rheumatoid arthritis, dermatomyositis/polymyositis, scleroderma, Sjögren syndrome, and Raynaud syndrome [4, 5, 10].

Adverse events may be delayed and may occur years after AM procedure [12].

### Exacerbation of rheumatic disease

According to the American Society for Dermatologic Surgery recommendations, dermal fillers are not recommended for patients with lupus or other connective tissue disorders. Data indicate the risk of cross-reactivity of anti-dsDNA antibodies with collagen; hence, collagen-based fillers should not be used in patients with systemic lupus erythematosus [29]. No evidence was found of sclerosis reactivation preceded by hyaluronic acid filling in cases of stable localized scleroderma [30]. Tattooing in psoriasis resulted in exacerbation in 1.3% of patients [16]

It is worth mentioning that withholding drugs before AM procedure has the potential to produce a risk of exacerbation.

### Drug interactions

Special care should be taken when using botulinum toxin (BTX) in patients taking immunosuppressive drugs [31, 32]. It should be avoided in patients treated with chloroquine, hydroxychloroquine, and cyclosporine [33].

## Summary

To date, the risk of adverse events associated with AM procedures in patients with AIRD was not estimated. There are studies regarding specific procedures in specific diseases (like tattooing in psoriasis or dermal fillers in scleroderma) [16, 30]. Systematic reviews are focused on procedures (like breast implants or dermal fillers) [34, 35]. A systematic review investigating adverse events of injectable fillers showed that events with poor prognosis are very rare, and the evidence certainty on the topic is low [34]. Further investigations in the field of AM and AIRD are needed. Thus, we recommend individual consulting before AM procedure with the rheumatologist to discuss the topics mentioned in Table 4.

**Table 4:** Key problems to discuss before esthetic procedure in patients with autoimmune inflammatory rheumatic diseases

Risk	Discuss possible adverse events Recommend special attention in perioperative period
Optimal time to perform procedure	Remission or low activity of AIRD
Drug assessment	Check for drug interactions If necessary, withhold therapy during the perioperative period

*AIRD* autoimmune inflammatory rheumatic disease

### Limitations

The number of patients in group 2 is relatively small, making it difficult to draw definite conclusions. Another important limitation lies in the fact that the information is based on patients’ reporting, not medical history. Rare and remote time adverse events may require that large cohort studies would be able to show potential correlation.

The strength of this survey is that it is the first such paper (after the PubMed literature review) that has attempted to evaluate the safety of AM procedures in patients treated with biologics.

## Conclusion

In our study, the use of esthetic medicine procedures in patients with AIRD, including those treated with bDMARDs, was associated with a risk of mild site reactions. Most of the patients expressed satisfaction with the results of the AM procedure and they would like to repeat the procedure in the future. AM procedure timing should be planned during remission or low disease activity. Patients undergoing immunosuppressive treatment need special attention to avoid wound infections.

### Supplementary Information

Below is the link to the electronic supplementary material.

Supplementary file1 (DOCX 22 KB)

## Data Availability

The authors confirm that the data supporting the findings of this study are available within the article and its supplementary materials.
