# Supplementary material for: Safety of esthetic procedures in rheumatic patients: single-center survey of patients
Source: Rheumatol Int. 2023 Oct 17;44(2):357–62. doi: 10.1007/s00296-023-05481-5 (PMC10796409; doi:10.1007/s00296-023-05481-5)
Supplement: Supplementary file 1 — Supplementary file1 (DOCX 22 KB) [file 296_2023_5481_MOESM1_ESM.docx]

Survey

**Use of aesthetic medicine procedures in patients with inflammatory rheumatic diseases**

*Dear ladies and gentlemen, we would like to ask you to complete this survey on the use of aesthetic medicine procedures. It will allow us to assess the safety of such procedures in patients with rheumatic diseases. Our survey is purely scientific, and all results that will be presented upon completion will be anonymous. We would also like to inform you that in accordance with Polish law, consent to participate in the study is voluntary.*

1. Sex m f
2. Age:
3. Education: Primary/secondary/higher
4. Place of residence: Rural locality/ city <100,00 inhabitants / city> 100,00 inhabitants
5. Diagnosed rheumatic disease and year of diagnosis

……………………………………………………………….

1. Other autoimmune disease (Hashimoto’s disease / Graves’ disease / skin psoriasis / Crohn’s disease / ulcerative colitis / sarcoidosis / multiple sclerosis / vitiligo / type 1 diabetes / Sjögren’s syndrome, other…………………………………………………….)
2. Other illness:
3. Type 2 diabetes
4. Tumor diagnosed within < 5 years; if yes, then what type…………………
5. Have you undergone aesthetic medicine/plastic surgery procedures?

Yes/ No

If yes, how many times?......................

1. Do you plan to undergo such procedures in the future?

Yes/ No

If so, will this be the first time/subsequent time? …………………….

1. What aesthetic medicine/plastic surgery procedures have you undergone? Please indicate the date of the procedure.
2. Tattoo Yes/ No Date……………………………………..

- Was red pigment used? Yes/ No Date..............................

1. Dermal fillers

- Hyaluronic acid Yes/ No Date …………………………
- Collagen Yes/ No Date …………………………
- Silicone Yes/ No Date …………………………
- Neuromodulators (botulinum toxin) Yes/ No Date ……..…
- Other Yes/ No Date …………………………

1. Plastic surgery Yes/ No

- What kind? ………………………………………………………..
- If a breast augmentation procedure was performed, what material was used (silicone inserts/saline) and what were the reasons for surgery (reconstruction after mastectomy/ cosmetic reasons) ……………………………………………

1. Piercing Yes/ No Data …………………………
2. Other ………………………………….. Yes/ No Data …………………………
3. At the time of the aforementioned procedure, the rheumatic disease was in your opinion:
4. Non-active
5. With a mild degree of activity
6. With a moderate degree of activity
7. With a high degree of activity
8. With a very high degree of activity
9. Were you using immunosuppressive drugs (including steroids)/biologics at the time of the above-mentioned procedure? If yes, which ones? ………………………………………………………………………..
10. Have you discontinued the medications listed in question 9 before the aforementioned procedure? If so, how many days prior? ...........................
11. Did you consult a physician (primary care/ rheumatologist) before performing the above-mentioned procedure?

Yes (primary care/rheumatologist) / No

1. Were the risks of possible complications discussed with you before performing the aforementioned procedure? Yes/ No
2. During the first two weeks after the procedure, did you observe an inflammatory reaction at the site of the performed procedure (swelling, pain, tenderness, redness, bruising, other)?

Yes / No

If so, what kind?………………………………………………………………………

If yes, has there been any intervention (consultation with a primary care physician/surgeon/dermatologist, consultation with the individual who performed the procedure, use of topical medications - ointments, therapeutic injections into lesions, use of oral medications - antibiotics, (steroids not due to a rheumatic disease))?

Yes/ No

What kind? …………………………………………………………………………

1. Have you observed any worrisome symptoms in the area of the procedure performed within >2 weeks, up to one year after the procedure, such as: ulcerations, inflammatory granulomas, acne lesions, other skin lesions with recurring infections (herpes, bacterial superinfections, hypertrophied scars, filler displacement, subcutaneous papules, others)?

Yes/ No

What kind? …………………………………………………………………………………………

If yes, has there been any intervention (consultation with a primary care physician/surgeon/dermatologist, consultation with the individual who performed the procedure, use of topical medications- ointments, injections into the lesions, use of oral medications- antibiotics, steroids (not due to rheumatic disease))?

…………………………………………………………………………………………

1. Have you observed any of the above-mentioned symptoms in the area of the procedure performed in the period > one year after the procedure?

Yes/ No

Which ones?.............................................................................................

1. Have you undergone a tattoo/implant/filler removal procedure? Yes/ No

If so, for what reason?

1. Medical reasons (local complications)
2. Medical reasons other…………………………………………
3. Non-medical reasons …………………………………………….
